# Supplementary material for: Xanthomonas Phage PBR31: Classifying the Unclassifiable
Source: Viruses. 2024 Mar 6;16(3):406. doi: 10.3390/v16030406 (PMC10975493; doi:10.3390/v16030406)
Supplement: Supplementary file 1 [file viruses-16-00406-s001.zip › Figure S1.zip!/Figure S1.pdf]

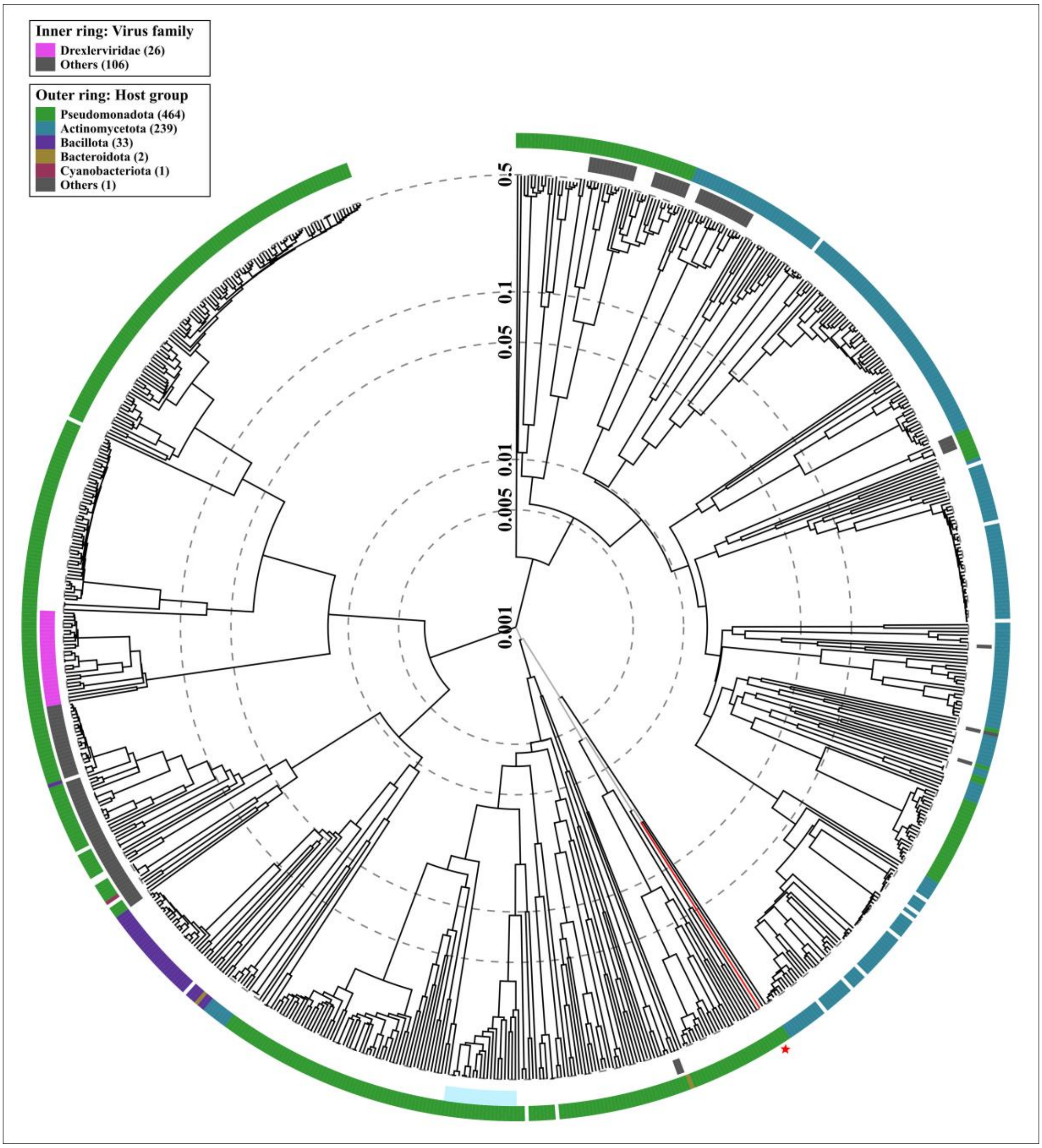

Inner ring: Virus family
Drexlerviridae (26)
Others (106)
Outer ring: Host group
Pseudomonadota (464)
Actinomycetota (239)
Bacillota (33)
Bacteroidota (2)
Cyanobacteriota (1)
Others (1)
0.5
0.1
0.05
0.01
0.005
0.001

(**a**)

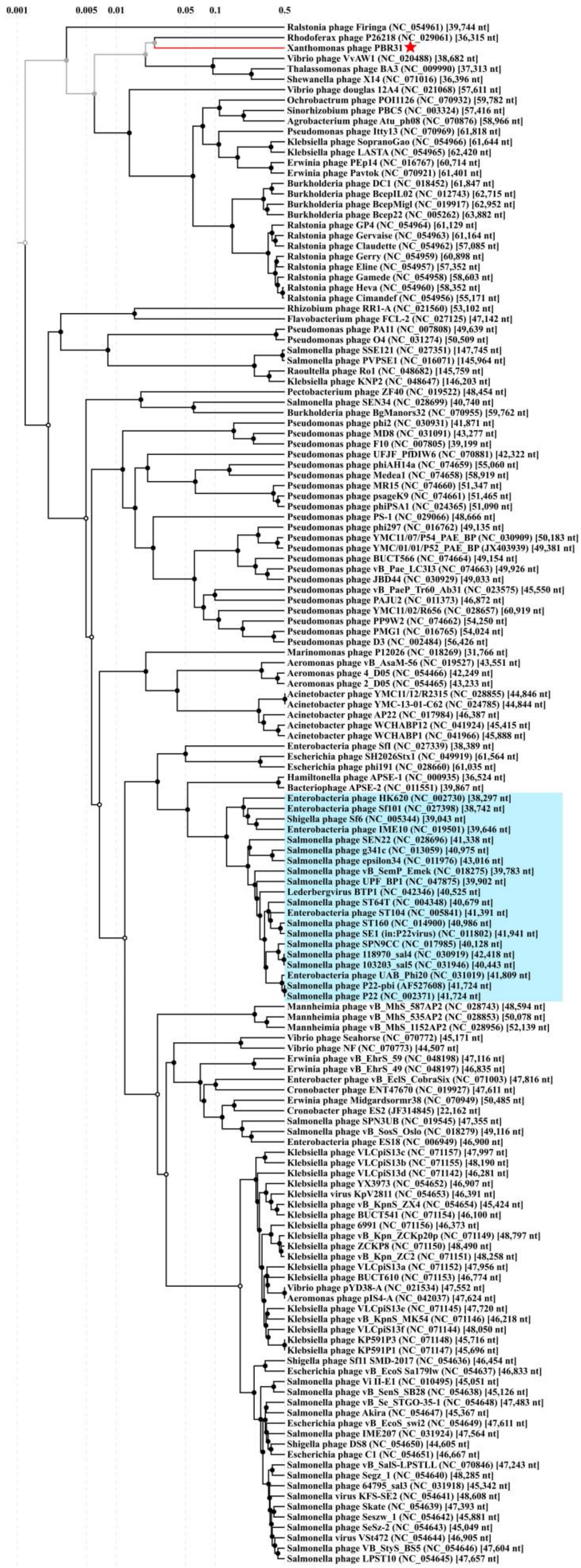
0.001
0.005
0.01
0.05
0.1
0.5
Ralstonia phage Firinga (NC_054961) [39,744 nt]
Rhodoferax phage P26218 (NC_029061) [36,315 nt]
Xanthomonas phage PBR31
Vibrio phage VvAW1 (NC_020488) [38,682 nt]
Thalassomonas phage BA3 (NC_009990) [37,313 nt]
Shewanella phage X14 (NC_071016) [36,396 nt]
Vibrio phage douglas 12A4 (NC_021068) [57,611 nt]
Ochrobactrum phage POI1126 (NC_070932) [59,782 nt]
Sinorhizobium phage PBC5 (NC_003324) [57,416 nt]
Agrobacterium phage Atu_ph08 (NC_070876) [58,966 nt]
Pseudomonas phage Itty13 (NC_070969) [61,818 nt]
Klebsiella phage SopranoGao (NC_054966) [61,644 nt]
Klebsiella phage LASTA (NC_054965) [62,420 nt]
Erwinia phage PEp14 (NC_016767) [60,714 nt]
Erwinia phage Pavtok (NC_070921) [61,401 nt]
Burkholderia phage DC1 (NC_018452) [61,847 nt]
Burkholderia phage BcepIL02 (NC_012743) [62,715 nt]
Burkholderia phage BcepMigl (NC_019917) [62,952 nt]
Burkholderia phage Bcep22 (NC_005262) [63,882 nt]
Ralstonia phage GP4 (NC_054964) [61,129 nt]
Ralstonia phage Gervaise (NC_054963) [61,164 nt]
Ralstonia phage Claudette (NC_054962) [57,085 nt]
Ralstonia phage Gerry (NC_054959) [60,898 nt]
Ralstonia phage Eline (NC_054957) [57,352 nt]
Ralstonia phage Gamede (NC_054958) [58,603 nt]
Ralstonia phage Heva (NC_054960) [58,352 nt]
Ralstonia phage Cimandef (NC_054956) [55,171 nt]
Rhizobium phage RR1-A (NC_021560) [53,102 nt]
Flavobacterium phage FCL-2 (NC_027125) [47,142 nt]
Pseudomonas phage PA11 (NC_007808) [49,639 nt]
Pseudomonas phage O4 (NC_031274) [50,509 nt]
Salmonella phage SSE121 (NC_027351) [147,745 nt]
Salmonella phage PVPSE1 (NC_016071) [145,964 nt]
Raoultella phage Ro1 (NC_048682) [145,759 nt]
Klebsiella phage KNP2 (NC_048647) [146,203 nt]
Pectobacterium phage ZF40 (NC_019522) [48,454 nt]
Salmonella phage SEN34 (NC_028699) [40,740 nt]
Burkholderia phage BgManors32 (NC_070955) [59,762 nt]
Pseudomonas phage phi2 (NC_030931) [41,871 nt]
Pseudomonas phage MD8 (NC_031091) [43,277 nt]
Pseudomonas phage F10 (NC_007805) [39,199 nt]
Pseudomonas phage UFJF_PfDIW6 (NC_070881) [42,322 nt]
Pseudomonas phage phiAH14a (NC_074659) [55,060 nt]
Pseudomonas phage Medea1 (NC_074658) [58,919 nt]
Pseudomonas phage MR15 (NC_074660) [51,347 nt]
Pseudomonas phage psageK9 (NC_074661) [51,465 nt]
Pseudomonas phage phiPSA1 (NC_024365) [51,090 nt]
Pseudomonas phage PS-1 (NC_029066) [48,666 nt]
Pseudomonas phage phi297 (NC_016762) [49,135 nt]
Pseudomonas phage YMC11/07/P54_PAE_BP (NC_030909) [50,183 nt]
Pseudomonas phage YMC/01/01/P52_PAE_BP (JX403939) [49,381 nt]
Pseudomonas phage BUCT566 (NC_074664) [49,154 nt]
Pseudomonas phage vB_Pae_LC3I3 (NC_074663) [49,926 nt]
Pseudomonas phage JBD44 (NC_030929) [49,033 nt]
Pseudomonas phage vB_PaeP_Tr60_Ab31 (NC_023575) [45,550 nt]
Pseudomonas phage PAJU2 (NC_011373) [46,872 nt]
Pseudomonas phage YMC11/02/R656 (NC_028657) [60,919 nt]
Pseudomonas phage PP9W2 (NC_074662) [54,250 nt]
Pseudomonas phage PMG1 (NC_016765) [54,024 nt]
Pseudomonas phage D3 (NC_002484) [56,426 nt]
Marinomonas phage P12026 (NC_018269) [31,766 nt]
Aeromonas phage vB_AsaM-56 (NC_019527) [43,551 nt]
Aeromonas phage 4_D05 (NC_054466) [42,249 nt]
Aeromonas phage 2_D05 (NC_054465) [43,233 nt]
Acinetobacter phage YMC11/12/R2315 (NC_028855) [44,846 nt]
Acinetobacter phage YMC-13-01-C62 (NC_024785) [44,844 nt]
Acinetobacter phage AP22 (NC_017984) [46,387 nt]
Acinetobacter phage WCHABP12 (NC_041924) [45,415 nt]
Acinetobacter phage WCHABP1 (NC_041966) [45,888 nt]
Enterobacteria phage SfI (NC_027339) [38,389 nt]
Escherichia phage SH2026Stx1 (NC_049919) [61,564 nt]
Escherichia phage phi191 (NC_028660) [61,035 nt]
Hamiltonella phage APSE-1 (NC_000935) [36,524 nt]
Bacteriophage APSE-2 (NC_011551) [39,867 nt]
Enterobacteria phage HK620 (NC_002730) [38,297 nt]
Enterobacteria phage Sf101 (NC_027398) [38,742 nt]
Shigella phage Sf6 (NC_005344) [39,043 nt]
Enterobacteria phage IME10 (NC_019501) [39,646 nt]
Salmonella phage SEN22 (NC_028696) [41,338 nt]
Salmonella phage g341c (NC_013059) [40,975 nt]
Salmonella phage epsilon34 (NC_011976) [43,016 nt]
Salmonella phage vB_SemP_Emek (NC_018275) [39,783 nt]
Salmonella phage UPF_BP1 (NC_047875) [39,902 nt]
Lederbergvirus BTP1 (NC_042346) [40,525 nt]
Salmonella phage ST64T (NC_004348) [40,679 nt]
Enterobacteria phage ST104 (NC_005841) [41,391 nt]
Salmonella phage ST160 (NC_014900) [40,986 nt]
Salmonella phage SE1 (in:P22virus) (NC_011802) [41,941 nt]
Salmonella phage SPN9CC (NC_017985) [40,128 nt]
Salmonella phage 118970_sal4 (NC_030919) [42,418 nt]
Salmonella phage 103203_sal5 (NC_031946) [40,443 nt]
Enterobacteria phage UAB_Phi20 (NC_031019) [41,809 nt]
Salmonella phage P22-pbi (AF527608) [41,724 nt]
Salmonella phage P22 (NC_002371) [41,724 nt]
Mannheimia phage vB_MhS_587AP2 (NC_028743) [48,594 nt]
Mannheimia phage vB_MhS_535AP2 (NC_028853) [50,078 nt]
Mannheimia phage vB_MhS_1152AP2 (NC_028956) [52,139 nt]
Vibrio phage Seahorse (NC_070772) [45,171 nt]
Vibrio phage NF (NC_070773) [44,507 nt]
Erwinia phage vB_EhrS_59 (NC_048198) [47,116 nt]
Erwinia phage vB_EhrS_49 (NC_048197) [46,835 nt]
Enterobacter phage vB_EclS_CobraSix (NC_071003) [47,816 nt]
Cronobacter phage ENT47670 (NC_019927) [47,611 nt]
Erwinia phage Midgardsormr38 (NC_070949) [50,485 nt]
Cronobacter phage ES2 (JF314845) [22,162 nt]
Salmonella phage SPN3UB (NC_019545) [47,355 nt]
Salmonella phage vB_SosS_Oslo (NC_018279) [49,116 nt]
Enterobacteria phage ES18 (NC_006949) [46,900 nt]
Klebsiella phage VLCpiS13c (NC_071157) [47,997 nt]
Klebsiella phage VLCpiS13b (NC_071155) [48,190 nt]
Klebsiella phage VLCpiS13d (NC_071142) [46,281 nt]
Klebsiella phage YX3973 (NC_054652) [46,907 nt]
Klebsiella virus KpV2811 (NC_054653) [46,391 nt]
Klebsiella phage vB_KpnS_ZX4 (NC_054654) [45,424 nt]
Klebsiella phage BUCT541 (NC_071154) [46,100 nt]
Klebsiella phage 6991 (NC_071156) [46,373 nt]
Klebsiella phage vB_Kpn_ZCKp20p (NC_071149) [48,797 nt]
Klebsiella phage ZCKP8 (NC_071150) [48,490 nt]
Klebsiella phage vB_Kpn_ZC2 (NC_071151) [48,258 nt]
Klebsiella phage VLCpiS13a (NC_071152) [47,956 nt]
Klebsiella phage BUCT610 (NC_071153) [46,774 nt]
Vibrio phage pYD38-A (NC_021534) [47,552 nt]
Aeromonas phage pIS4-A (NC_042037) [47,624 nt]
Klebsiella phage VLCpiS13e (NC_071145) [47,720 nt]
Klebsiella phage vB_KpnS_MK54 (NC_071146) [46,218 nt]
Klebsiella phage VLCpiS13f (NC_071144) [48,050 nt]
Klebsiella phage KP591P3 (NC_071148) [45,716 nt]
Klebsiella phage KP591P1 (NC_071147) [45,696 nt]
Shigella phage Sf11 SMD-2017 (NC_054636) [46,454 nt]
Escherichia phage vB_EcoS Sa179lw (NC_054637) [46,833 nt]
Salmonella phage Vi II-E1 (NC_010495) [45,051 nt]
Salmonella phage vB_SenS_SB28 (NC_054638) [45,126 nt]
Salmonella phage vB_Se_STGO-35-1 (NC_054648) [47,483 nt]
Salmonella phage Akira (NC_054647) [45,367 nt]
Escherichia phage vB_EcoS_swi2 (NC_054649) [47,611 nt]
Salmonella phage IME207 (NC_031924) [47,564 nt]
Shigella phage DS8 (NC_054650) [44,605 nt]
Escherichia phage C1 (NC_054651) [46,667 nt]
Salmonella phage vB_SalS-LPSTLL (NC_070846) [47,243 nt]
Salmonella phage Segz_1 (NC_054640) [48,285 nt]
Salmonella phage 64795_sal3 (NC_031918) [45,342 nt]
Salmonella virus KFS-SE2 (NC_054641) [48,608 nt]
Salmonella phage Skate (NC_054639) [47,393 nt]
Salmonella phage Seszw_1 (NC_054642) [45,881 nt]
Salmonella phage SeSz-2 (NC_054643) [45,049 nt]
Salmonella virus VSt472 (NC_054644) [46,905 nt]
Salmonella phage VB_StyS_BS5 (NC_054646) [47,604 nt]
Salmonella phage LPST10 (NC_054645) [47,657 nt]

(**b**)

Figure S1. (**a**) Circular proteomic dendrogram plotted by ViPTree using the genome of PBR31 and genomes found by ViPTree to be related to the PBR31 genome. (**b**) The clade of the proteomic tree containing PBR31 phages and *Lederbervirus* phages. *Xanthomonas* phage PBR31 is marked with a red asterisk and *Lederbervirus* phages are highlighted in blue. The scale shows the number of estimated substitutions per site.
